# Engineered Matrices Enable the Culture of Human Patient‐Derived Intestinal Organoids

**DOI:** 10.1002/advs.202004705

**Published:** 2021-03-12

**Authors:** Daniel R. Hunt, Katarina C. Klett, Shamik Mascharak, Huiyuan Wang, Diana Gong, Junzhe Lou, Xingnan Li, Pamela C. Cai, Riley A. Suhar, Julia Y. Co, Bauer L. LeSavage, Abbygail A. Foster, Yuan Guan, Manuel R. Amieva, Gary Peltz, Yan Xia, Calvin J. Kuo, Sarah C. Heilshorn

**Affiliations:** ^1^ Department of Chemical Engineering Stanford University Stanford CA 94305 USA; ^2^ Department of Stem Cell Biology and Regenerative Medicine Stanford University Stanford CA 94305 USA; ^3^ Department of Bioengineering Stanford University Stanford CA 94305 USA; ^4^ Department of Materials Science & Engineering Stanford University Stanford CA 94305 USA; ^5^ Department of Chemistry Stanford University Stanford CA 94305 USA; ^6^ Department of Medicine and Hematology Stanford University School of Medicine Stanford CA 94305 USA; ^7^ Department of Pediatrics (Infectious Diseases) and of Microbiology and Immunology Stanford University Stanford CA 94305 USA; ^8^ Department of Anesthesiology Stanford University School of Medicine Stanford CA 94305 USA

**Keywords:** 3D cell culture, adult stem cells, engineered biomaterial, extracellular matrix, intestinal organoid

## Abstract

Human intestinal organoids from primary human tissues have the potential to revolutionize personalized medicine and preclinical gastrointestinal disease models. A tunable, fully defined, designer matrix, termed hyaluronan elastin‐like protein (HELP) is reported, which enables the formation, differentiation, and passaging of adult primary tissue‐derived, epithelial‐only intestinal organoids. HELP enables the encapsulation of dissociated patient‐derived cells, which then undergo proliferation and formation of enteroids, spherical structures with polarized internal lumens. After 12 rounds of passaging, enteroid growth in HELP materials is found to be statistically similar to that in animal‐derived matrices. HELP materials also support the differentiation of human enteroids into mature intestinal cell subtypes. HELP matrices allow stiffness, stress relaxation rate, and integrin‐ligand concentration to be independently and quantitatively specified, enabling fundamental studies of organoid–matrix interactions and potential patient‐specific optimization. Organoid formation in HELP materials is most robust in gels with stiffer moduli (*G’* ≈ 1 kPa), slower stress relaxation rate (*t*_1/2_ ≈ 18 h), and higher integrin ligand concentration (0.5 × 10^−3^–1 × 10^−3^ m RGD peptide). This material provides a promising in vitro model for further understanding intestinal development and disease in humans and a reproducible, biodegradable, minimal matrix with no animal‐derived products or synthetic polyethylene glycol for potential clinical translation.

## Graphical abstract

A tunable, designer matrix, termed hyaluronan elastin‐like protein (HELP) that enables the formation, differentiation, and passaging of adult primary tissue‐derived organoids is reported. HELP matrices allow stiffness, stress relaxation rate, and integrin‐ligand concentration to be independently and quantitatively specified, enabling fundamental studies of organoid–matrix interactions and potential patient‐specific optimization.

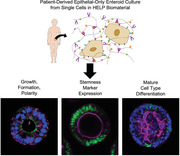

## 1 Introduction

Human intestinal organoids derived from primary tissue of patient biopsies have the potential to revolutionize personalized medicine and preclinical models of human gastrointestinal disease.^[^ ^1^, ^2^, ^3^ ^]^ Most intestinal organoids are grown in a decellularized matrix derived from Engelbreth‐Holm‐Swarm (EHS) mouse sarcoma, (e.g., Matrigel or Cultrex).^[^ ^4^, ^5^ ^]^ Unfortunately, these materials have  limited tunability and reproducibility.^[^ ^6^ ^]^ In response, synthetic matrices have been designed to support the formation of murine and human induced pluripotent stem cell (iPSC)‐derived intestinal organoids without requiring EHS matrix or other cell types.^[^ ^7^, ^8^, ^9^, ^10^, ^11^ ^]^ In contrast, human patient‐derived intestinal organoids (pIOs) in synthetic matrices have often required either a spheroid formation step in EHS matrix or co‐culture with mesenchymal cells.^[^ ^7^, ^12^ ^]^ Recent work to develop engineered matrices for pIOs have relied on polyethylene glycol (PEG) as a synthetic matrix backbone,^[^ ^13^, ^14^ ^]^ although PEG is known to interact with the immune system and induce antibody formation.^[^ ^15^, ^16^ ^]^ Furthermore, due to its high swelling ratio, PEG is often incompatible with microfluidic devices for lab‐on‐a‐chip applications.^[^ ^17^ ^]^ To create a PEG‐free system, we report a designer matrix, hyaluronan elastin‐like protein (HELP), that enables the formation, differentiation, and passaging of adult primary tissue‐derived, epithelial‐only intestinal organoids. Three critical variables of HELP (matrix stiffness, matrix stress relaxation rate, and matrix integrin‐ligand concentration) can each be independently and quantitatively specified, enabling fundamental studies of organoid–matrix interactions and potential patient‐specific optimization. Our material provides a promising, 3D in vitro model for further understanding intestinal development and enteric disease in humans and a reproducible, biodegradable, minimal matrix with no animal‐derived products or synthetic PEG for potential clinical translation.

## 2 Results and Discussion

**Figure 1:**
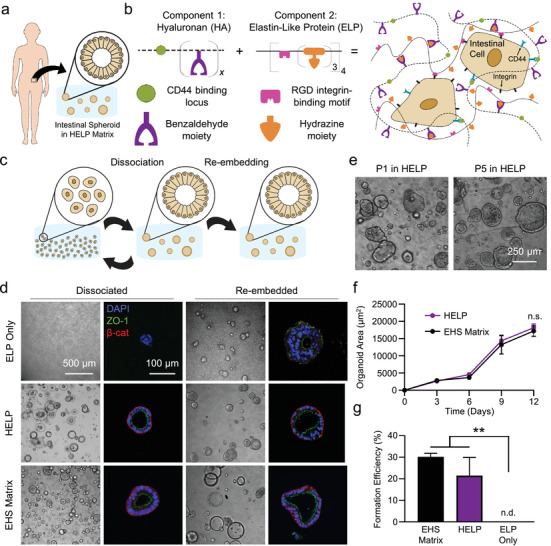
HELP matrix for patient‐derived intestinal organoid formation, growth, and passaging. a) Enteroids were generated from intestinal tissue biopsies from human patients. b) Schematic of HELP matrix, which is composed of benzaldehyde‐modified hyaluronan (HA) and hydrazine‐modified elastin‐like protein (ELP). Hyaluronan can engage the CD44 receptors on cells, while recombinant ELP contains an RGD peptide ligand that engages cell integrin receptors. c) Schematic of enteroid passaging techniques. Enteroids can either be dissociated into single cells or directly re‐embedded as fully formed enteroids into a new material at the time of passaging. d) Brightfield and confocal fluorescence micrographs of enteroids in different materials, when dissociated (left) and re‐embedded (right) in these materials. e) Representative brightfield images of enteroids grown on Passage 1 and Passage 5 in HELP, with dissociation into single cells at the time of each passage. f) Growth curves of dissociated enteroids grown from Passage 12 in EHS matrix or Passage 12 in HELP; at each timepoint, a two‐tailed Student's *t*‐test was conducted, *n* = 3, n.s. = not significant. g) Enteroid formation efficiency for enteroids grown from single cells in EHS matrix, HELP, or ELP only. One‐way ANOVA with Tukey's multiple comparisons testing; ** = *p* < 0.01, *n* = 3, n.d. = none detected. All data shown are mean ± SD.

We hypothesized that a minimal matrix inspired by biopolymers found in the native intestine would support the formation of organoids derived from primary human tissue (**Figure** **1**a). Our group previously reported a protein‐engineered matrix that supported the formation and growth of primary murine intestinal organoids using amino acid sequences derived from human elastin and fibronectin.^[^ ^9^ ^]^ Elastin is one of the major components of the intestinal extracellular matrix,^[^ ^18^ ^]^ and fibronectin is expressed within the intestinal stem cell (ISC) crypt.^[^ ^19^ ^]^ Our recombinant elastin‐like protein (ELP, *M*_W_ 37.7 kDa) intersperses elastin‐like sequences with an integrin‐binding, extended RGD sequence borrowed from fibronectin (Figure 1b and Figure S1a, Supporting Information). In vivo, ISC maintenance and proliferation is mediated in part by the CD44 receptor, with CD44 activation associated with intestinal growth.^[^ ^20^, ^21^ ^]^ The CD44 receptor can interact with hyaluronan (HA), a glycosaminoglycan important for normal intestinal growth,^[^ ^22^, ^23^, ^24^, ^25^ ^]^ leading us to hypothesize that an engineered matrix that included HA could support pIO cultures. To create reproducible hydrogel materials from these two biopolymer components, both the HA (*M*_W_ 100 kDa) and ELP are recombinantly synthesized. Several recombinantly synthesized materials have gained widespread clinical use, suggesting that this synthetic strategy may have clinical potential.^[^ ^26^ ^]^ After recombinant synthesis of the individual components, HELP gels are formed with straightforward bioconjugation reactions. Specifically, ELP is modified with hydrazine groups, as previously reported,^[^ ^27^ ^]^ and we developed a new strategy to chemically modify HA with benzaldehyde functional groups (Figure S1b–e, Supporting Information).^[^ ^28^, ^29^ ^]^ Simply mixing the two modified biopolymers together induced the formation of hydrazone bonds^[^ ^30^ ^]^ resulting in a hydrogel network we term HELP.

Throughout this work, we classify cells in the following way: we refer to undifferentiated spheroids of intestinal epithelial cells as enteroids; upon differentiation, we term these intestinal cell structures organoids, or patient‐derived intestinal organoids (pIOs). In some work, what we term the enteroid state is referred to as an ISC colony or spheroid, in particular in murine systems where fluorescent reporter lines for Lgr5^+^ ISC populations are used.^[^ ^7^ ^]^ Here, human, patient‐derived enteroids were dissociated into single cells and embedded within HELP during covalent crosslinking of the hydrogel. Within 3 days, de novo spheroid formation was observed (Figure 1c,d). Some previous reports of pIOs in synthetic materials require an initial step of enteroid formation in EHS matrix before encapsulation in synthetic biomaterials,^[^ ^7^ ^]^ a process we term “re‐embedding” (Figure 1c). Here, we compared the ability of different materials to support these two distinct culture methods: 1) encapsulation of dissociated single cells and 2) re‐embedding of pre‐formed enteroids (Figure 1d). As expected and consistent with previous reports,^[^ ^7^ ^]^ re‐embedded enteroids were able to grow and remain viable for at least 6 days within ELP‐only matrices that contain the fibronectin‐derived, integrin‐binding RGD ligand, although we note that enteroid polarization, as observed by *β*‐catenin staining, was not maintained (Figure 1d, top). In stark contrast, dissociated human intestinal cells within the ELP‐only matrix (i.e., without HA) were unable to form enteroids, suggesting that the RGD ligand alone is insufficient to support de novo pIO formation in this material. Interestingly, adding HA to the engineered matrix enabled single intestinal cells to robustly form enteroids in HELP, as well as survive at least 6 days when cultured as re‐embedded enteroids. Importantly, enteroids formed in HELP exhibited proper intestinal epithelial polarity, similar to EHS matrix, as indicated by the localization of ZO‐1 tight junction protein to the apical lumen and by basolateral localization of the adherens junction protein *β*‐catenin (Figure 1d). In addition to proper polarization, HELP gels supported high rates of viability (≥90%) post‐seeding at multiple cell densities (Figure S2, Supporting Information). Similar results were observed for a second, distinct patient line within HELP matrices (Figure S3a, Supporting Information). To demonstrate the potential broad applicability of HELP matrices to support organoid growth, primary murine intestinal enteroids and human iPSC‐derived hepatic organoids were also viable in HELP matrices (Figure S4, Supporting Information).

In addition to robust enteroid formation from single cells, enteroids within HELP can be repeatedly passaged, and continue to form new enteroids after each passage (Figure 1e and Figures S3c and S5, Supporting Information). HELP matrices are enzymatically degraded using elastase and hyaluronidase, followed by enteroid dissociation into single cells with trypsin (see the Experimental Section). Encapsulation of these single cells into fresh HELP matrix resulted in successful new enteroid formation for up to 12 passages without visible change in enteroid morphology (Figure 1e,f). To investigate potential changes in gene expression upon repeated passaging in HELP, we compared expression of known intestinal stem cell markers at early (P2) and late (P10) passages within HELP to that in EHS matrix (Figure S6a, Supporting Information). We observed no statistically significant changes in the expression levels of the genes *LGR5* (leucine‐rich repeat‐containing G‐protein coupled receptor 5), *BMI1* (B lymphoma Mo‐MLV insertion region 1 homolog), and *SOX9* (SRY‐Box transcription factor 9), suggesting that repeated passages maintain a stem‐like quality. Consistent with this interpretation, enteroids that had been passaged repeatedly in HELP matrices were able to form enteroids that matched the growth rate of those in EHS matrix, as observed by brightfield microscopy over 12 days of culture (Figure 1f). Furthermore, these repeatedly passaged enteroids had statistically similar formation rates in HELP and EHS matrices (Figure 1g).

**Figure 2:**
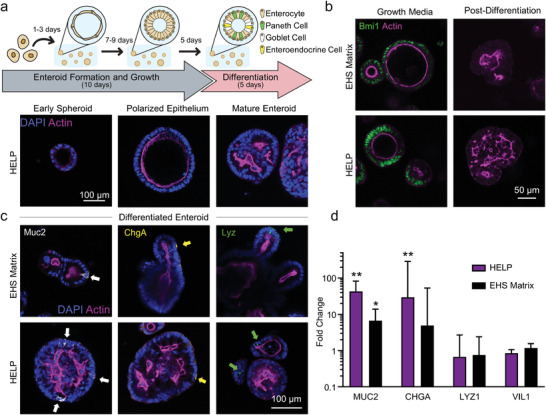
Differentiation of organoids grown in HELP and EHS matrices. a) Top: Schematic of differentiation experiment timeline. Organoids are cultured from single cells in growth medium for 10 days, followed by 5 days in differentiation medium (see the Experimental Section). Bottom: Confocal micrographs illustrating the progression of organoids from early enteroid to polarized enteroid to differentiated organoid. b) Confocal micrographs demonstrating the observance (left) and absence (right) of intestinal stem cell marker Bmi1 during culture in growth and differentiation media, respectively. c) Confocal micrographs demonstrating the observance of mature intestinal cell subtypes: Paneth cells (Lyz^+^, left), goblet cells (Muc2^+^, middle), and enteroendocrine cells (ChgA^+^, right). d) RT‐qPCR quantification of changes in RNA expression of differentiated cell type markers, compared to cells that were maintained for 15 days in maintenance medium, and relative to control gene BACT. Under the assumption that *C*_T_ values are normally distributed, two‐tailed Student's *t*‐tests were performed on *C*_T_ values between differentiated versus maintenance cultures for each gene in each material, respectively. ** = *p* < 0.01, * = *p* < 0.05, *N* = 3 independent experiments, *n* = 4 technical replicates. Data shown are geometric mean ± geometric SD.

To assess whether HELP could support differentiation of pIOs, single cells embedded in HELP were first allowed to form enteroids for 10 days in growth medium, containing Wnt3A, epidermal growth factor, Noggin, and R‐spondin1 (**Figure** **2**a). While in growth medium, cells undergo initial enteroid formation (Figure 2a, bottom left). As the enteroids develop, the cells became columnar in morphology and adopt native intestinal apicobasal polarity, demonstrated by the thick apical actin border within the organoid (Figure 2a, bottom center), localization of known polarization markers (Figure 1d), and expression of the known intestinal stem cell marker Bmi1 (Figure 2b, left). On day 10, Wnt and R‐spondin1 were removed to promote enteroid differentiation into organoids over 5 days. This process resulted in the formation of undulating lumens (Figure 2a, bottom right, Video S1, Supporting Information) and a decrease in Bmi1 expression (Figure 2b, right). This morphology is commonly observed in primary human intestinal enteroids and is qualitatively different from the budding “crypt‐like” architecture observed in murine intestinal organoids.^[^ ^4^, ^13^, ^31^ ^]^

After differentiation, immunocytochemistry confirmed chromogranin A (ChgA), a marker of differentiated enteroendocrine cells both in HELP and EHS matrices (Figure 2c). Higher expression of mucin‐2 (Muc2)‐positive goblet cells was observed in organoids grown in HELP compared to those grown in EHS matrix. Lysozyme‐positive Paneth cells were observed in organoids grown both in HELP and in EHS matrices. To assess the transcriptional expression of these differentiation markers, reverse transcription quantitative polymerase chain reaction (RT‐qPCR) was performed on organoids differentiated in HELP or EHS matrices compared to enteroids that were maintained in growth medium in their respective matrices for 15 days (Figure 2d). A high upregulation of *CHGA* (chromogranin A) and *MUC2* (mucin‐2) gene expression was observed in both HELP and EHS matrix. *LYZ1* (lysozyme) and enterocyte marker *VIL1* (villin‐1) expression levels were relatively unchanged compared to the undifferentiated controls in both HELP and EHS matrix. To investigate this further, we performed immunostaining on enteroids pre‐differentiation and found the presence of lysozyme‐positive cells and villin‐positive cells in both HELP and EHS matrix (Figure S6b, Supporting Information). Thus, consistent with reports from others, the pre‐differentiation growth conditions used for these studies results in some spontaneous differentiation of Paneth cells and enterocytes.^[^ ^32^, ^33^ ^]^ Interestingly, upon repeat passaging, we observed that the spontaneous gene expression of lysozyme, as well as mucin‐2, was significantly decreased in pre‐differentiated enteroids in HELP compared to EHS matrix (Figure S6c, Supporting Information), while the expression of the intestinal stem cell marker proteins was unchanged (Figure S6a, Supporting Information).

**Figure 3:**
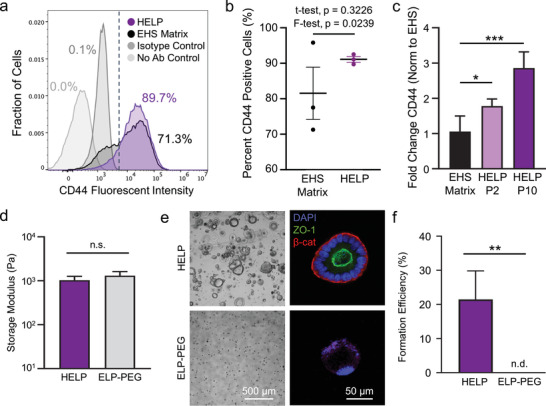
Role of hyaluronan in HELP matrices. a) Flow cytometric analysis of enteroids grown in EHS and HELP matrices 11 days post‐encapsulation for CD44‐positive cells, compared to negative controls, dashed line indicates flow gating. b) Quantification of percentages of CD44‐positive cells in EHS matrix and HELP across three independent experiments. c) Gene expression of CD44 for passage 20 enteroids cultured in HELP for different lengths of time and EHS matrix (all three conditions had 20 total passages, normalized to P20 in EHS). One‐way ANOVA with Tukey post‐hoc testing, *n* = 4, * = *p* < 0.05, *** = *p* < 0.001. d) Storage moduli for HELP and ELP‐PEG formulations as measured by oscillatory rheology. At least three measurements were performed for each material. Two‐tailed Student's *t*‐test, n.s. = not significant. e) Brightfield and confocal micrographs at 6 days post‐seeding of enteroids grown in HELP and ELP‐PEG matrices, where HA is absent in the ELP‐PEG gels. f) Formation efficiency between enteroids grown in HELP and ELP‐PEG. Two‐tailed Student's *t*‐test, ** = *p* < 0.01, *n* = 3, n.d. = none detected. Data shown are mean ± SD.

Because human enteroids were successfully formed in HELP but not ELP‐only matrices (Figure 1), we next sought to further explore the permissive role of HA in these matrices. To confirm that patient‐derived intestinal cells present CD44, which is enriched in proliferative intestinal crypts and is also an HA receptor,^[^ ^21^, ^22^, ^23^, ^24^, ^25^ ^]^ we performed flow cytometry on single dissociated cells from undifferentiated enteroids grown in HELP compared to those grown in EHS matrices (**Figure** **3**a,b and Figure S7, Supporting Information). Flow cytometric analysis of three different experimental repeats revealed that about 90% of cells from enteroids grown in HELP were consistently CD44 positive (Figure 3b). In contrast, the percentage of CD44‐positive cells from enteroids grown in EHS matrix was 71%, 78%, and 96% across three different trials. These repeats were performed at both earlier and late passages (P14 and P18 in EHS and P3, P4, and P8 in HELP). While the means of these data were not statistically different (*t*‐test *p*‐value = 0.3226), the standard deviation of these data suggests that EHS matrix results in significantly more variability in CD44 presentation (*F*‐test, *p* = 0.0239). These data are consistent with reports that EHS matrix can have significant batch to batch variability.^[^ ^6^ ^]^ Interestingly, at the RNA level, we observed that repeat passaging in HELP appears to increase CD44 expression compared to passaging in EHS matrix (Figure 3c).

Taken together, these results suggest that HA signaling in HELP may play an important role in promoting enteroid formation. To further test whether HA signaling was a key feature of our material that assisted in enteroid formation, we modified a synthetic PEG polymer with the same benzaldehyde moiety that we used to modify our HA component. Using this material, we generated an ELP‐PEG hydrogel matrix that was stiffness‐matched to the HELP matrix (Figure 3d), with an equivalent concentration of RGD peptide (1 × 10^−3^ m). Brightfield and confocal fluorescence microscopy revealed that enteroids were unable to form in ELP‐PEG gels of equivalent mechanical properties to HELP, suggesting that HA biochemical signaling is a required component of the HELP matrix (Figure 3e,f). Consistent with our data, previously reported PEG‐based biomaterials for epithelial‐only, intestinal organoids also found that the RGD ligand alone was insufficient to support differentiation.^[^ ^7^, ^13^ ^]^ Interestingly, in these previous reports, alternative biochemical supplements were identified to the HA used here (recombinant laminin‐111 for murine cultures and a combination of GFOGER integrin‐binding peptides with matrix‐binding peptides for murine and human cultures). Thus, an emerging body of literature is beginning to suggest that a more complex biochemical signaling microenvironment beyond the RGD ligand may be required for in vitro differentiation of human organoids.

**Figure 4:**
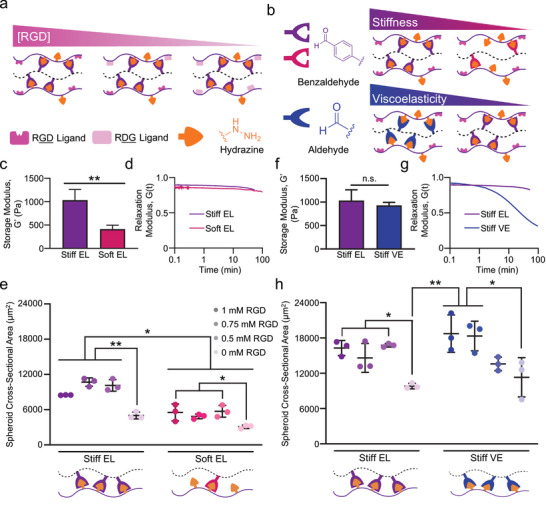
Custom tailoring of HELP matrix properties. a) HELP schematic illustrating specification of the RGD ligand concentration by blending different ELP variants in the material. b) HELP schematic illustrating that matrix stiffness is precisely modulated by changing the number of crosslinks (top), while matrix viscoelasticity is tuned by replacing benzaldehyde with aldehyde moieties. c) Shear storage moduli of Stiff Elastic (EL) and Soft EL HELP formulations. Student's *t*‐test, ** = *p* < 0.01, *N* = 3–5. d) Step‐strain–stress relaxation curves comparing EL formulations. e) Cross‐sectional area measurements of enteroids in EL materials at 12 days post‐encapsulation as single cells. Two‐way ANOVA with Tukey multiple comparisons testing, * = *p* < 0.05, ** = *p* < 0.01, *n* = 3. f) Shear storage moduli of Stiff EL and Stiff Viscoelastic (VE) formulations. Student's *t*‐test, *N* = 3–5, n.s. = not significant. g) Step‐strain–stress relaxation curves comparing Stiff EL and Stiff VE formulations. h) Cross‐sectional area measurements of enteroids in stiff materials at 12 days post‐encapsulation as single cells. Two‐way ANOVA with Tukey multiple comparisons testing, * = *p* < 0.05, ** = *p* < 0.01, *n* = 3. All data shown are mean ± SD.

The HELP material allows independent selection of multiple biomaterial properties, allowing the study of organoid growth in response to different biochemical and biophysical matrix cues. Tuning these parameters allows the careful study of how cells in pIOs respond to tissue mechanics. Indeed, the interplay of matrix stiffness, matrix stress relaxation, and matrix RGD content has been important in other engineered biomaterial systems.^[^ ^27^, ^30^, ^34^ ^]^ To explore these interactions within the HELP system, we first prepared a variant of our ELP protein that contains a nonintegrin binding, sequence‐scrambled peptide (RDG) that is known to be noncell‐adhesive.^[^ ^35^ ^]^ Next, by blending together RGD‐ and RDG‐ELP proteins within the HELP matrix, we were able to tune the exact concentration of RGD ligands in our hydrogels from 0 × 10^−3^ to 1 × 10^−3^ m without impacting the matrix mechanics (**Figure** **4**a and Figure S8, Supporting Information). To create matrices with a range of mechanical properties, we first varied the degree of hydrazine‐benzaldehyde crosslinking (Figure 4b, top) to create a stiff, elastic matrix (storage modulus, *G’* ≈ 1 kPa, termed “Stiff EL”) and a more compliant, elastic matrix (*G’* ≈ 400 Pa, “Soft EL”) (Figure 4c,d). These matrices had equivalent, quasi‐elastic stress relaxation profiles, with negligible stress relaxation over 60 min and a stress‐decay half‐time (*t*_1/2_) of about 18 h.

We measured the distributions of enteroid sizes as a function of varying material properties in sets of HELP matrices (Figure S9, Supporting Information). In general, the cross‐sectional area of enteroids grown in stiffer gels (≈1 kPa) was larger than those grown in softer gels (≈400 Pa, Figure 4e). This result is consistent with the stiffness range previously found to be optimal for murine as well as primary human intestinal organoids grown in synthetic PEG‐based matrices.^[^ ^7^, ^13^, ^14^ ^]^ In addition to this trend, we observed that enteroids did not grow as robustly in matrices lacking the RGD ligand, independent of matrix stiffness. This is consistent with previous reports of intestinal organoids in synthetic matrices, where minimum threshold levels of RGD were required for optimum organoid growth.^[^ ^7^, ^8^ ^]^ However, in these previous reports, organoids cultured in synthetic matrices without RGD were unable to form and remain viable. The fact that viable enteroids were able to form in 0 × 10^−3^ m RGD HELP matrices suggests that signaling from HA may be sufficient to induce some organoid formation, although co‐presentation of RGD and HA results in improved organoid growth.

We next explored the role of RGD concentration within matrices that were stress relaxing and viscoelastic. A matrix that is “stress relaxing” will undergo molecular‐level remodeling to dissipate and relieve the stress after it has been deformed by cellular forces.^[^ ^36^, ^37^ ^]^ Work with other cell types has demonstrated that matrix stress relaxation may have even stronger effects on cell phenotype than matrix stiffness, and these effects vary with the integrin ligand concentration, since this is how cells exert forces onto the matrix.^[^ ^38^, ^39^, ^40^, ^41^ ^]^ Thus, we sought to design a family of biomimetic HELP matrices that enabled independent tuning of matrix stiffness, RGD ligand concentration, and matrix stress relaxation. A key feature of native extracellular matrices and EHS matrices is their ability to undergo stress relaxation due to their physical crosslinks, which can easily be remodeled.^[^ ^34^ ^]^ The remodeling kinetics of dynamic covalent crosslinks, such as those used in our HELP matrices, can be tuned through selection of the neighboring chemical moieties.^[^ ^42^ ^]^ By replacing a fraction of the benzaldehyde groups on HA with aldehyde groups (Figure 4b, bottom), we formulated a faster stress relaxing, viscoelastic HELP matrix (termed “Stiff VE”) with an identical stiffness as the quasi‐elastic “Stiff EL” HELP matrix and a *t*_1/2_ ∼ 30 min (Figure 4f,g). This control over stress relaxation rate was not possible in our previously reported hydrazone‐crosslinked gels.^[^ ^29^ ^]^ Although stress relaxation rate is important for studying tissue‐specific cell behavior, it has been historically challenging to study due to confounding changes in other material properties.^[^ ^43^ ^]^ Interestingly, we observed a greater dependence on RGD concentration in the Stiff VE gels, with a higher threshold between 0.5 × 10^−3^ and 0.75 × 10^−3^ m RGD required for robust enteroid growth (Figure 4h). These data suggest that in matrices capable of undergoing greater remodeling, enteroids may be more sensitive to the presence of RGD ligand and integrin‐based adhesion, while dissociated cells can more robustly form enteroids across a broader range of matrix properties in matrices with more elastic‐like mechanics.

Interested in quantifying the change in matrix mechanical properties over the nearly 2 weeks culture, we utilized a microrheological technique based on dynamic light scattering (DLS) in order to measure the change in the storage moduli of EHS and HELP matrices over time in culture.^[^ ^44^ ^]^ We encapsulated primary human intestinal epithelial cells in hydrogels in the presence of PEGylated nanoparticles (500 nm) within plastic cuvettes suitable for analysis by DLS. When we measured the change in rheological properties within EHS matrix, as well as Stiff EL and Stiff VE HELP hydrogels with growing, encapsulated enteroids within them, we observed no statistically significant change in matrix properties throughout the 12 days culture period (Figure S10, Supporting Information). This suggests that no drastic change in matrix mechanics over time was responsible for the enteroid growth and formation that we observe in these engineered hydrogels. We note, however, that DLS microrheology is a technique that relies on spatial averaging, and thus does not exclude the possibility of significant matrix softening at the periphery of the enteroids. An interesting future direction would be to further evaluate potential local changes in the matrix biochemistry and biomechanics during extended culture.

## 3 Conclusion

In summary, here we present the HELP matrix that allows for the robust formation, growth, passaging (Figure 1), and differentiation (Figure 2) of primary, human intestinal organoids from dissociated single cells. Interestingly, the presence of hyaluronan within HELP is sufficient to enable de novo enteroid formation from single cells, as ELP‐only and ELP‐PEG matrices with similar mechanics and RGD‐ligand concentration did not support enteroid formation (Figures 1 and 3). We correlate this observation with an increase in expression of CD44, a well‐known receptor for HA, in enteroids cultured in HELP matrices (Figure 3). This receptor is known to be important in ISC renewal, thus our findings contribute to the collective understanding of matrix factors impacting intestinal cell proliferation.^[^ ^20^, ^21^ ^]^

The HELP matrix is not only suitable for this specific cell source, but also supports murine intestinal organoids as well as epithelial organoids from hepatocytes (Figure S4, Supporting Information). These results, combined with the bespoke tailoring of several material properties, including biochemical ligand density, matrix stiffness, and matrix stress relaxation rate (Figure 4), position the HELP matrix as a platform that can be optimized for the culture of a wide variety of patient‐derived epithelial organoids. In the future, the HELP matrix could be customized to mimic patient‐specific matrix properties, resulting in reproducible, personalized organoid cultures. HELP matrices add to the emerging library of minimal matrices available for reproducible organoid culture. Our well‐defined, minimal, engineered matrix overcomes the key limitations of EHS matrices, notably batch‐to‐batch variability, insufficient tunability, biological complexity, and lack of clinical translatability.^[^ ^6^ ^]^ Previous work has demonstrated that elastin‐like proteins and hyaluronic acid materials are well tolerated in vivo both individually and when combined to form hydrogels.^[^ ^45^, ^46^, ^47^, ^48^, ^49^ ^]^ An important next step toward clinical translation of cell therapies cultured in this material would be to test the immunogenicity of HELP in vivo. By enabling the culture of human intestinal and other epithelial organoids, the HELP material has numerous future applications, including in studies of enteric disease pathology, developmental biology, and personalized medicine.

## 4 Experimental Section

##### Human Organoid Passaging and Maintenance Culture in EHS Matrix

Human primary intestinal tissues were obtained from the Stanford Tissue Bank. Procedures for generation of human enteroid lines from patient tissue samples were approved by the Stanford University Medical Center (SUMC) Institutional Review Board and performed under protocol #28908. Written informed consent for research was obtained from donors prior to tissue acquisition. Enteroids were used between passages 4 and 24 for all experiments. Cells in maintenance cultures were maintained while encapsulated in 40 µL of EHS matrix, specifically Cultrex Basement Membrane Extract‐Reduced Growth Factor (BME‐RGF) Type 2 (Trevigen, Gaithersburg, MD) within 24‐well plates. Organoids were passaged every 1–2 weeks depending on growth rate. To passage organoids, Cultrex droplets were flooded with ice‐cold, 5 × 10^−3^ m ethylenediamine tetraacetic acid (EDTA) in phosphate buffered saline (PBS) to dissolve the gel, centrifuged for 5 min at 500 *x g*, treated with TrypLE (Thermo Fisher Scientific, Waltham, MA) for 10 min at 37 °C, with vigorous mixing by pipette aspiration every 5 min to assist in the generation of single cells. The TrypLE was then quenched with organoid growth medium (described below), and centrifuged for 5 min at 500 *x g*. The pellet was washed in growth medium for cell counting, and then centrifuged for 5 min at 500 x *g*. The cell pellet was resuspended in ice‐cold Cultrex at a concentration of 750 000 cells mL^−1^ and transferred to the cell culture incubator. After 10 min of gelation at 37 °C, 500 µL of pre‐warmed organoid growth medium was added to each well. Small molecule inhibitors, 10 × 10^−6^ m Y‐27632 and 2.5 × 10^−6^ m CHIR‐99021 (both obtained from Bio‐Techne, Minneapolis, MN), were added to the medium for the first media change of maintenance cultures. Media was completely replaced every 3–4 days.

##### Mouse Organoid Isolation and Culture

Murine intestinal organoids were generated as described previously.^[^ ^4^ ^]^ Briefly, isolated murine small intestines were transected in the longitudinal direction, and washed with cold PBS. The tissue was then minced into roughly 5‐mm square pieces and washed again with cold PBS. Tissue was then incubated in 2 × 10^−3^ m EDTA in PBS on ice. Following this incubation, the EDTA solution was then aspirated, and tissue fragments were then mixed well with a 10 mL serological pipette using cold PBS, and the tissue was allowed to settle. The supernatant was discarded, and the sediment containing intestinal crypts was resuspended in PBS. Samples were vigorously mixed and then centrifuged for 5 min at 500 *x g*, and then the crypt‐rich supernatant was then passed through a 70 µm cell strainer (BD Biosciences, San Jose, CA). Crypts were centrifuged once more for 3 min at 200 *x g* to separate out single cells. The fraction of mostly pure crypts was then used for culture.

##### Human Hepatic Organoid Isolation and Culture

Procedures for obtaining human primary tissue for the purpose of generating iPSC‐derived hepatic organoid lines were approved by the SUMC Institutional Review Board according to protocol #10368. Written informed consent for research was obtained from donors prior to tissue acquisition. Hepatic organoids were generated as described previously.^[^ ^50^ ^]^ Briefly, normal iPSC‐derived secondary hepatic organoid (HO2) were digested in 0.25% trypsin‐EDTA for 5–10 min. The cells were collected by centrifugation at 200 *g* for 3 min, resuspended in 25 µL of 1% HA, and directly mixed with preloaded 25 µL 1% ELP at 1000 cells per well in a 24‐well plate. After HA‐ELP solidification, 1 mL of growth media was added, and the cells were cultured for 6 days. The growth media was consisted of RPMI plus B27 (Thermo Fisher Scientific, Waltham, MA) medium with the following growth factors: 250 × 10^−9^ m LDN‐193189, 3 × 10^−6^ m CHIR99021, 10 × 10^−6^ m A83‐01, 100 ng mL^−1^ EGF, 10 ng mL^−1^ FGF10, and 20 ng mL^−1^ HGF. The cells were then cultured for 6 more days in a differentiation medium, which was consisted of HCM (Lonza, Basel, SUI) medium supplemented with 10 × 10^−6^ m DAPT, 10 ng mL^−1^ oncostatin M, 20 ng mL^−1^ HGF, 10 × 10^−6^ m dexamethasone, and 10 ng mL^−1^ BMP4. To perform forskolin‐induced swelling assay on HOs, both forskolin (FSK) and 3‐isobutyl‐1‐methylxanthine (IBMX) (10 × 10^−6^ m and 100 × 10^−6^ m, respectively) were added to activate cAMP pathway and increase CFTR function in HOs culture for 24 h. Swelling was visualized after staining with a 10 × 10^−6^ m solution of the cell‐permeable fluorescent dye calcein green. The difference in total area of each hepatic organoid after 24 h treatment was then calculated and plotted.

##### Organoid Growth Medium Generation

Organoid growth base media was consisted of a 1:1 mixture of ADMEM‐F12 media (Thermo Fisher Scientific, Waltham, MA) and L‐WRN (ATCC CRL3276) conditioned media. To generate L‐WRN conditioned media, L‐WRN cells were plated on T150 cell culture flasks in L‐WRN growth medium (Dulbecco's modified essential medium (DMEM) supplemented with 10% fetal bovine serum (FBS) and 1% penicillin‐streptomycin‐glutamine (PSQ)) and allowed to grow for 1–2 days. Growth media was changed and supplemented with L‐WRN selection medium (L‐WRN growth medium supplemented with 500 µg mL^−1^ each of G418 and hygromycin antibiotics to select for cells containing transgenic DNA encoding the secretion of Wnt‐3A, R‐spondin 3, and Noggin). Cells were grown until confluent, and split at a 1:4 ratio twice, and then split into multiple T150 flasks. Cells were cultured in L‐WRN growth medium until confluent, washed with L‐WRN collection medium (ADMEM‐F12 with 10% FBS and 1% PSQ), and cultured for 24 h with fresh L‐WRN collection medium. After 24 h, conditioned media was recovered from each flask and combined. Fresh L‐WRN collection medium was replaced, and conditioned media generation and collection was repeated up to four times. ADMEM‐F12 was mixed 1:1 with L‐WRN conditioned media and supplemented with the following reagents: 1 × 10^−3^ m HEPES (4‐(2‐hydroxyethyl)‐1‐piperazineethanesulfonic acid, Thermo Fisher Scientific, Waltham, MA), 1x Glutamax (Thermo Fisher Scientific, Waltham, MA), 10 × 10^−3^ m nicotinamide (Sigma‐Aldrich, St. Louis, MO), 1 × 10^−3^ m *N*‐acetylcysteine (Sigma‐Aldrich, St. Louis, MO), 1x B‐27 supplement (Thermo Fisher Scientific, Waltham, MA), 0.5 × 10^−6^ m A83‐01 (Sigma‐Aldrich, St. Louis, MO), 1x PSQ (Thermo Fisher Scientific, Waltham, MA), 10 × 10^−9^ m Gastrin‐I (Sigma‐Aldrich, St. Louis, MO), 10 × 10^−6^ m SB‐202190 (Bio‐Techne, Minneapolis, MN), 50 ng mL^−1^ recombinant EGF (Thermo Fisher Scientific, Waltham, MA), and 1x Normocin (InvivoGen, San Diego, CA).

##### Organoid Differentiation

In order to differentiate enteroids into organoids, cells were maintained in growth medium for 10 days after encapsulation as single cells in HELP or EHS matrices, washed briefly with PBS, and switched into differentiation medium for 5 days of culture. Differentiation medium was Advanced DMEM/F12 medium supplemented with 1x Glutamax, 1x Penicillin/Streptomycin, 1x Normocin, 100 ng mL^−1^ recombinant noggin (Peprotech, Rocky Hill, NJ), 1x B27, 1 × 10^−3^ m *N*‐acetylcysteine, 50 ng mL^−1^ recombinant EGF, 10 × 10^−9^ m gastrin‐I, 10 × 10^−6^ m Y‐27632 ROCK inhibitor, 5 × 10^−6^ m DAPT, and 500 × 10^−9^ m A83‐01.

##### ELP‐Hydrazine Synthesis

ELP was prepared as described previously.^[^ ^35^, ^51^ ^]^ Briefly, ELP sequences were cloned into pET15b plasmids, and a T7 promoter was used to control protein expression. BL21(DE3)pLysS *Escherichia coli* (Life Technologies) containing ELP‐encoding plasmids were cultured in Terrific Broth to an OD_600_ of 0.8, and 1 × 10^−3^ m isopropyl *β*‐D‐1‐thiogalactopyranoside (IPTG) was used to induce expression. Bacteria were allowed to express protein for 7 h, and were subsequently harvested by centrifugation, suspended in TEN buffer (10 × 10^−3^ m Tris, 1 × 10^−3^ m EDTA, and 100 × 10^−3^ m NaCl, pH 8.0), and lysed via three cycles of freeze–thaw. Cell lysate was treated with deoxyribonuclease (DNase) and 1 × 10^−3^ m phenylmethanesulfonyl fluoride (PMSF) to inhibit proteolysis. ELP was purified by an alternating sequence of centrifugation steps at 4 and 37 °C, followed by dialysis against deionized (DI) water for four shifts (48 h, 4 L volume per shift), then frozen at −80 °C, and lyophilized. To modify ELP amines with hydrazine functional groups, lyophilized ELP (210 mg) was completely dissolved at 7 wt% in 3 mL of anhydrous dimethyl sulfoxide (DMSO) and then diluted to 3.5 wt% with 3 mL of anhydrous *N,N*‐dimethylformamide (DMF). In a round‐bottom flask, 3 mL of anhydrous DMF was used to separately dissolve tri‐Boc‐hydrazinoacetic acid (2 equiv:ELP amine), hexafluorophosphate azabenzotriazole tetramethyl uronium (HATU, 2 equiv:ELP amine), and 4‐methylmorpholine (4.5 equiv:ELP amine), and this vessel was stirred for 5 min to allow HATU to activate the free acids on the tri‐Boc‐hydrazinoacetic acid. Next, the ELP solution was added to the round‐bottom flask dropwise with stirring. The reaction was allowed to proceed overnight at room temperature (RT). The product was precipitated in ice‐cold ether, centrifuged, and dried, yielding the Boc‐protected ELP‐hydrazine intermediate. This intermediate was analyzed by ^1^H NMR to quantify the modification efficiency (500 Hz, DMSO‐*d*_6_) *δ* 7.00 (d, 2H), 6.62 (d, 2H), 1.46 (m, 27H). Modification efficiency was determined by comparing the integrated signal of the Boc protons (*δ* 1.5–1.35) to the aromatic protons of tyrosine residues on ELP (*δ* 7.00 and 6.62). To remove the Boc protecting groups, the ELP‐hydrazine intermediate was dissolved at 2 wt% in 1:1 DCM:TFA with 2.5% v/v triisopropylsilane and stirred at RT for 4 h in a vented round‐bottom flask. The product was precipitated in ether, centrifuged, and dried, then dissolved in DI water and dialyzed against DI water for three shifts (24 h, 4 L volume per shift), sterile filtered using a 0.2 µm filter, and lyophilized.

##### Hyaluronic Acid Modification

100 kDa sodium hyaluronate (HA, Lifecore Biomedical, Chaska, MN, USA) was modified to have an aldehyde functional group by the following overall procedure: first the carboxylic acid groups on HA were amidated with propargylamine, generating an HA‐alkyne intermediate; then, copper click chemistry was used to react this alkyne with the azide moiety of a heterobifunctional small molecule containing an aldehyde functional group onto the HA, generating HA functionalized with aldehydes.

*HA‐alkyne of 12% modification*: HA was dissolved in 2‐(*N*‐morpholino)ethanesulfonic acid (MES) buffer (0.2 m, pH 4.5) to a concentration of 10 mg mL^−1^. To this solution, *N*‐hydroxysuccinimide (NHS, 0.8 eq. to the HA dimer unit), 1‐ethyl‐3‐(3‐dimethylaminopropyl) carbodiimide (EDC, 0.8 eq.), and propargyl amine (0.8 eq.) were added successively. After adjusting pH to 6, the mixture was stirred at RT for 4 h. The solution was then dialyzed against DI water for six shifts (3 days, 4 L volume per shift) and lyophilized to give a white powder.

*HA‐alkyne of 30% modification*: Sodium hyaluronate was dissolved in MES buffer (0.2 m, pH 4.5) to a concentration of 10 mg mL^−1^. To this solution, NHS (1.5 eq. to the HA dimer unit), EDC (1.5 eq.), and propargyl amine (1.0 eq.) were added successively. After adjusting pH to 6, the mixture was stirred at RT for 4 h. The solution was then dialyzed against DI water for six shifts (3 days, 4 L volume per shift) and lyophilized to give a white powder.

HA‐alkynes were then modified by the following small molecule, 1, to generate HA‐benzaldehyde. The small molecule was generated as follows:

2

*N*‐(2‐azidoethyl)‐4‐formylbenzamide (**1**) was synthesized according to the method published previously.^[^ ^28^ ^]^ HA was modified with molecule **1** according to previously reported procedure with minor modifications.^[^ ^28^ ^]^ HA‐alkyne (300 mg) was dissolved in PBS at 2 wt% followed by the addition of **1** (1 eq. to HA dimer unit). A minimal amount of DMSO was used to dissolve **1** before it was added to the HA solution. The solution was then bubbled with N_2_ for 30 min. Copper (II) sulfate pentahydrate (0.004 eq.) and sodium ascorbate (0.06 eq.) were dissolved in DI water, bubbled with N_2_, and added to the HA solution. After stirring at RT for 1 day, the mixture was dialyzed against DI water for 3 days and lyophilized. Since the proton signals of aromatic rings on the benzaldehyde moiety overlap with triazole groups, the degree of modification on HA‐benzaldehyde was quantified by integration of the proton signal (*δ* = 7.5–8, 5H) relative to that of the methyl groups on *N*‐acetylglucosamine of HA backbone (*δ* = 1.8, 3H).

*HA‐aldehyde synthesis*: HA‐aldehyde was synthesized according to a method published previously.^[^ ^52^ ^]^ HA was first dissolved at 0.4 w/v% in Milli‐Q water while stirring at room temperature. An aqueous solution of 0.1 m sodium periodate was added dropwise, and the reaction was stirred overnight at room temperature in the dark. The following day, ethylene glycol was added for 1 h to inactivate any unreacted periodate. The solution was then purified by dialysis with a 10 000 MWCO membrane against Milli‐Q water for 3 days, with fresh water changed in shifts of 12 h. After dialysis, the solution was sterile filtered using a 0.2 µm filter, and the product was obtained via freeze‐drying.

##### PEG‐Benzaldehyde (PEG‐BZA) Synthesis

PEG‐BZA was synthesized as previously described.^[^ ^30^ ^]^ Briefly, 4‐formyl benzoic acid (0.528 g, 3.52 mmol, 2.1 eq. per amine; Sigma) was dissolved in 5 mL anhydrous DMF (Sigma) and activated with HATU (1.216 g, 3.2 mmol, 2 eq.; Sigma) and 4‐methylmorpholine (0.792 mL, 7.2 mmol, 4.5 eq.; Sigma). The reaction was allowed to stir for 5 min before the addition of 4‐arm 10 kDa PEG‐amine (4 g, 0.2 mmol; Creative PEGworks) dissolved in 5 mL DMF for a total reaction volume of 10 mL. The reaction was allowed stir at RT overnight. The final polymer was precipitated in ethyl ether (Thermo Fisher), pelleted by centrifugation at 22 000 rcf for 20 min, and re‐dissolved in Milli‐Q water. PEG‐BZA was dialyzed (MWCO: 3500 Da; Spectrum) against Milli‐Q water for 3 days at 4 °C, and dialysis water was changed two to three times per day. PEG‐BZA was lyophilized and stored at −20 °C. Modification of PEG‐BZA was estimated using ^1^H NMR (500 MHz). PEG‐BZA was dissolved in deuterated water (D_2_O; Sigma) at 10 mg mL^−1^. *δ* = 9.9 ppm (1H, s, aldehyde); *δ* = 7.93 and 7.82 ppm (2H each; d; benzene ring); *δ* = 3.56 (217H per arm; s; PEG).

##### Engineered Hydrogel Formation and Rheological Characterization

Mechanical testing was performed on a stress‐controlled ARG2 rheometer (TA) using a 20 mm diameter, 1° cone‐plate geometry with a 28 µm gap between the geometry and the rheometer stage. The two hydrogel components were dissolved separately at 2 wt% in PBS and kept on ice. First, 25 µL of the HA gel component was pipetted onto the middle of the rheometer stage, then 25 µL of the ELP component was pipetted directly into the droplet of HA, and the pipette tip was used to mix the components together. The rheometer head was promptly lowered, and the hydrogel components were allowed to react under 1 Hz, 1% strain oscillatory shear for 10 min at RT and 5 min at 37 °C. This protocol was immediately followed by a frequency sweep from 0.1 to 10 Hz at 1% strain. The storage and loss moduli were taken to be the value at 1 Hz from these measurements. For stress relaxation measurements, samples were allowed to gel in situ for 10 min at RT and 10 min at 37 °C under 1 Hz, 1% oscillatory shear, and then a 5% step strain was applied. The stress relaxation response was measured for at least 45 min. The *t*_1/2_ for each material was calculated as the time at which the stress had decayed to 50% of its stabilized initial value. Measurements were taken in at least triplicate.

##### Cell Encapsulation within Engineered Matrices

To form cell‐laden HELP hydrogels, ELP and HA gel components were separately dissolved at 2 wt% in PBS. To generate dissociated cultures, cells were passaged as described above, and the pellet was suspended in the ELP component and kept on ice. 3 µL of a selected HA gel component was added to the bottom of a 6 µL silicone mold (4 mm diameter, 0.5 mm height, plasma bonded to a 12 mm circular #1 coverglass). Then, 3 uL of the ELP‐cell solution was pipetted directly into the droplet of HA. The pipette tip was then used to mix the two hydrogel components and homogenously disperse the cells within the hydrogel. Hydrogels were allowed to crosslink for 10 min at RT and 10 min at 37 °C, after which 750 µL of growth medium was added. To form ELP‐only gels, unmodified ELP protein was dissolved in PBS at a concentration of 3.25 w/v% at 4 °C. A 5x solution of crosslinker tetrakis(hydroxymethyl)phosphonium chloride (THPC) was prepared by diluting 1:750 in PBS. Cells were passaged as described above, and the pellet was resuspended in the unmodified ELP component and kept on ice. ELP solution was then mixed with THPC at a 4:1 ELP:THPC volume ratio and mixed well by pipette aspiration before pipetting the ELP‐THPC mixture into silicone‐glass molds. ELP‐only cultures were allowed to crosslink for 15 min at RT, followed by 15 min at 37 °C. For ELP‐PEG gels, enteroids were passaged as described above, and single cells were suspended in 4 w/v% ELP‐Hydrazine on ice. 3 µL of 8 w/v% PEG‐BZA component was then pipetted onto the bottom of 6 µL silicone‐glass molds while plates were kept on ice. 3 µL of ELP component with cells was then added to the PEG component and mixed by swirling with the pipette tip. These gels were then allowed to crosslink for 1 h at 4 °C, followed by 15 min at RT and 15 min at 37 °C, followed by the addition of 750 µL of pre‐warmed growth medium. For re‐embedded cultures, enteroids in EHS matrices were incubated with 5 × 10^−3^ m EDTA on ice for 45–60 min to completely dissociate the matrix, then centrifuged for 5 min at 500 x *g*. Cells were then washed with growth medium and again centrifuged for 5 min at 500 x *g*. To approximately keep cell seeding density consistent, cell counts from equivalent maintenance culture wells that had been dissociated into single cells were always conducted, and the assumption that equivalent volumes of maintenance culture had approximately equivalent numbers of cells was made to allow control of re‐embedded enteroid seeding density. Growth medium was changed every 3–4 days. For all experiments, small molecule inhibitors Y‐27632 and CHIR‐99021 were not included in the media like they were for EHS maintenance cultures.

##### Cell Viability Measurements

Primary human intestinal epithelial cells were encapsulated in EHS and HELP matrices as described above at three different cell densities into 6 µL hydrogels: 375, 750, and 1500 cells per µL. After hydrogel crosslinking, hydrogels were submerged in 750 uL of a mixture of 2 × 10^−6^ m calcein AM (Thermo Fisher Scientific, Waltham, MA) and 4 × 10^−6^ m ethidium homodimer (Thermo Fisher Scientific) in PBS for 20 min at 37 °C in a cell culture incubator. Samples were washed with PBS before imaging of samples was performed by confocal fluorescence microscopy. Tile‐scanned z‐stacks were performed on each of three replicates per condition to allow quantification of all cells from the hydrogels. Images were taken no more than 1 h after the removal of staining solution from the hydrogels. To process images and quantify viability, a custom ImageJ macro was written to threshold maximum projection images at a fixed pixel intensity level. Binary images were then generated from the thresholded images, and processed using the “Fill Holes” command, followed by the “Watershed” command. Live and dead cells were, respectively, quantified using the “Analyze Particles” command, with a minimum circularity of 0.4, and a size range between 100 and 500 µm^2^ used to filter out noise and large aggregates.

##### Enteroid Passaging in HELP Matrices

HELP‐maintained cultures were generated from cells that had been previously passaged in EHS matrix 8–11 times. Maintenance cultures of 10–40 µL of HELP matrix were generated in silicone molds, as described above. Enteroids in HELP were passaged every 10–14 days. To passage enteroids in HELP, the matrix was first degraded with 100 U mL^−1^ elastase from porcine pancreas (Thermo Fisher Scientific, Waltham, MA) and 2500 U mL^−1^ hyaluronidase from bovine testes (Sigma‐Aldrich, St. Louis, MO) dissolved in PBS. Culture medium was completely aspirated from culture wells, including on the upper surface of the silicone molds. Once this upper surface was dried, a droplet of elastase‐hyaluronidase mixture equal to the gel volume was added on top of the gel. The gels were incubated at 37 °C for 1 h to allow for complete matrix degradation. Enteroids were then pipetted into a 15 mL conical centrifuge tube in an excess of growth medium to dilute the enzymes. Enteroids were spun down for 5 min at 500 x *g*, and the pellet was then washed with growth medium and centrifuged once more for 5 min at 500 x *g*. Enteroids were then passaged as described above, and single cells were encapsulated in HELP as described above.

##### Enteroid Formation and Growth Analysis

To analyze organoid formation efficiency, up to 100 organoids were analyzed per gel for three separate gels per condition. Within 4–6 h after encapsulation in EHS matrix or HELP materials, the initial cell culture is observed under brightfield microscopy to ensure the presence of only single cells. Every 3 days, brightfield images of each well were taken at 10x magnification. For every well at each time point, three fields of view were chosen, and three z‐slices were taken in every field of view. To analyze enteroid growth, a Wacom Intuos tablet was used to trace the outlines of enteroids and quantify enteroid size using the Particle Analysis feature in FIJI (ImageJ, NIH). A enteroid formation threshold of 2000 µm^2^ was selected based on previously reported enteroid morphology. A morphological criterion was also applied to separate viable enteroids from those that were severely misshapen. From this analysis, enteroid sizes and counts were collected for each well. Using the size of each image, and an ≈250 µm z‐volume for the three z‐slices, organoid formation efficiency for each well was calculated by extrapolating the organoid count per z‐stack volume in each well and comparing it to the initial cell seeding density, assuming uniform cell distribution. Formation efficiency for each condition was then calculated as an average of the three wells. To calculate average organoid size, distribution statistics were generated for the pooled three wells. Outliers were excluded from data sets as follows

(1) $$\mathrm{Outlier}>1.5\times \left(Q3-Q1\right)+Q3$$

where

*Q*3= 3rd quartile of data

*Q*1= 1st quartile of data

Average organoid cross‐sectional area was then calculated as the average of the three wells per condition.

##### Immunocytochemistry

Cells are fixed and stained within the hydrogel. To prepare samples for fixation, each well was washed briefly with pre‐warmed PBS. Cells were fixed by adding 750 µL of pre‐warmed 4% paraformaldehyde (PFA) with 0.1% glutaraldehyde in PBS and incubating at 37 °C for 30–45 min. Fixation solution was then aspirated and three 5 min washes of PBS were performed. Cells were permeabilized for 30 min with 0.25% v/v Triton X‐100 in PBS (PBST), then blocked for 3 h in PBS with 5 wt% bovine serum albumin (BSA), 5% v/v goat serum, and 0.5% v/v Triton X‐100. Primary antibody dilutions (see Table S1, Supporting Information) were prepared in PBS with 2.5 wt% BSA, 2.5% v/v goat serum, and 0.5% v/v Triton X‐100 (Antibody Dilution Solution), and primary incubation was performed overnight at 4 °C. Antibody solutions were removed, and three 5 min washes in PBST were performed. Secondary antibodies were diluted 1:500 in Antibody Dilution Solution and incubated overnight at 4 °C. Secondary antibody solution was then removed and washed twice with PBST for 30 min each. 1:2000 dilution of DAPI and 1:250 dilution of phalloidin were prepared in PBST and incubated for 45 min, followed by three 5 min washes of PBST. Samples were then dried of excess liquid and inverted onto a droplet of ProLong Gold Antifade mounting medium on top of a rectangular coverglass. Mountant was allowed to cure for 48 h in the dark at RT before imaging on a DMI4000 B confocal microscope (Leica, Wetzlar, Germany).

##### Quantitative Real‐Time RT‐PCR Analysis

Hydrogels were removed from silicone molds using a pipette tip to scrape and transfer the gels into 1.5 mL Eppendorf tubes containing 500 µL of Trizol reagent (Invitrogen, Carlsbad, CA) on ice to extract RNA. The solution was then sonicated to allow complete break‐up of hydrogels for optimal RNA extraction. Phenol‐chloroform extraction was used to isolate RNA with Phase Lock Gels (Quantabio, Beverly, MA). A constant amount of RNA (0.1–1 µg) was reverse transcribed using the High‐Capacity cDNA Reverse Transcription Kit (Applied Biosystems, Foster City, CA). 1 µg of cDNA in 5 µL of nuclease free water was then mixed with 10 µL of Fast SYBR Green Master Mix (Applied Biosystems, Foster City, CA) and run on the Applied Biosystems StepOnePlus Real Time PCR System. Primers used in this work are listed in Table S2 in the Supporting Information.

##### Flow Cytometric Analysis

Organoids were dissociated into single cells following the methods outlined above (see Human Organoid Passaging and Maintenance Culture in EHS Matrix and Enteroid Passaging in HELP Matrices). The cells were centrifuged for 5 min at 500 *x g* to pellet. The media was then removed from each pellet and the cells were resuspended in FACS buffer (PBS + 1 × 10^−3^ m EDTA (Invitrogen) + 2% v/v FBS (Atlanta Bio) + 1% penicillin/streptomycin (Gibco)) supplemented with fluorophore‐conjugated primary antibodies (BioLegend anti‐human CD44 antibody, BioLegend IgG2B isotype control). Antibody staining was performed for 30 min at 4 °C in the dark. Following staining, the cells were washed twice using FACS buffer and resuspended in 200 µL FACS buffer with DAPI (1:10000, BioLegend) to select for live cells. Flow cytometry was performed on a Beckman Coulter CytoFlex analyzer (Stanford Stem Cell Institute FACS Core). To analyze the data, gates were determined using forward and side scatter with height and width used to identify cell doublets. Subsequently, live DAPI‐negative cells were gated for all marker analyses and population frequency calculations.

##### Preparation of Functionalized Nanoparticles for DLS

To measure matrix mechanics across the duration of culture, passivated polystyrene nanoparticles were required in order to produce light scattering without chemically interacting with matrices or media components. To synthesize passivated polystyrene nanoparticles, 500 nm carboxylated polystyrene beads (Polysciences, Warren, PA) were suspended in 50 × 10^−3^ m MES buffer at pH 6.0 at 1.3% w/v. The beads were then reacted in the presence of 2 × 10^−3^ m 1‐ethyl‐3‐(3‐dimethylaminopropyl)carbodiimide (EDC, Sigma) and 5 × 10^−3^ m sulfo‐*N*‐hydroxysuccinimide (sulfo‐NHS, Sigma) for 30 min at room temperature under gentle rotation. After this activation step, the resulting solution was further reacted in the presence of 1 × 10^−3^ m of 2 kDa polyethylene glycol diamine (PEG‐DA, Sigma) for 30 min at room temperature under gentle rotation. Beads were pelleted by centrifugation at 9000 x *g* for 3 min and washed repeatedly with Milli‐Q water before use, and final reconstitution was at 0.26% w/v. Final bead size was confirmed using a Zetasizer Nano ZS instrument (Malvern Panalytical, Malvern, UK).

##### DLS Microrheology

To prepare functionalized beads for cell encapsulations, a solution of beads was incubated in an antibiotic cocktail consisting of penicillin, streptomycin, amphotericin B, and Normocin at 4 °C overnight. Beads were pelleted and washed as above three times prior to diluting at 1:10 in respective HELP gel components. Plastic cuvettes (BrandTech Scientific, Essex, CT) and caps were sterilized by soaking in 70% ethanol overnight followed by drying on the day of the encapsulation. Primary human intestinal epithelial cells were encapsulated in HELP and EHS matrices as described above, and 40 µL gels were cast into the bottoms of cuvettes, and crosslinked as described above, with special care given to avoid bubble formation which could affect measurements. Microrheological characterization was performed using a Zetasizer Nano ZS (Malvern Panalytical, Malvern, UK) with a custom analysis playlist. Rheological data were extracted from the autocorrelation functions of the particle scattering intensity for each sample, and were converted into storage moduli using a custom analysis package in Python.

##### Statistical Analysis

The following statistical significance representation was used for all significance testing in this publication: *,^#^ = *p* < 0.05, ** = *p* < 0.01, *** = *p* < 0.001, **** = *p* < 0.0001. Data from Figures 1g and 3d, and Figure S3 in the Supporting Information were analyzed by one‐way analysis of variance (ANOVA) with Tukey post‐hoc testing to compare individual means. The data from Figure 2d were analyzed via unpaired, two‐tailed Student's *t*‐test to compare gene expression changes between undifferentiated and differentiated conditions for each material. The data from Figure 4c,f were analyzed using a two‐tailed Student's *t*‐test. The data from Figure 4e,h were analyzed by two‐way ANOVA with Tukey post‐hoc testing to compare individual means. The data in Figure S7 in the Supporting Information were analyzed using a Kruskal–Wallis test with Dunn's multiple comparison test. All statistical analysis was performed using GraphPad Prism 8.0 software (GraphPad Software, La Jolla, CA, USA).

## Conflict of Interest

The authors declare no conflict of interest.

## Supporting information

Supporting Information

Supplemental Video 1
