# Supplementary material for: Engineered Matrices Enable the Culture of Human Patient‐Derived Intestinal Organoids
Source: Adv Sci (Weinh). 2021 Mar 12;8(10):2004705. doi: 10.1002/advs.202004705 (PMC8132048; doi:10.1002/advs.202004705)
Supplement: Supplementary file 1 — Supporting Information [file ADVS-8-2004705-s001.pdf]

Supplemental Information

Engineered matrices enable the culture of human patient-derived intestinal organoids

Daniel R. Hunt, Katarina C. Klett, Shamik Mascharak, Huiyuan Y. Wang, Diana Gong, Junzhe Lou, Xingnan Li, Pamela C. Cai, Riley A. Suhar, Julia Y. Co, Bauer L. LeSavage, Abbygail A. Foster, Yuan Guan, Manuel R. Amieva, Gary Peltz, Yan Xia, Calvin J. Kuo, Sarah C. Heilshorn

Contact information: heilshorn@stanford.edu

List of Supplemental Information:

Figure S1. HELP materials characterization
Figure S2. Cell viability upon 3D cell encapsulation
Figure S3. Human intestinal organoids robustly form in HELP in more than one patient-derived cell line
Figure S4. HELP supports mouse intestinal and human hepatic organoid formation and growth
Figure S5. Intestinal organoids can be passaged within EHS or HELP
Figure S6. Expression of intestinal markers over time in HELP
Figure S7. Flow cytometric analysis for $CD44^+$ cells in enteroids grown in HELP and EHS
Figure S8. Oscillatory rheology on various materials
Figure S9. Enteroid growth distributions across HELP formulations
Figure S10. DLS Microrheology to measure matrix mechanics over time

Supplemental Video S1. Confocal microscopy of intestinal organoid in HELP.

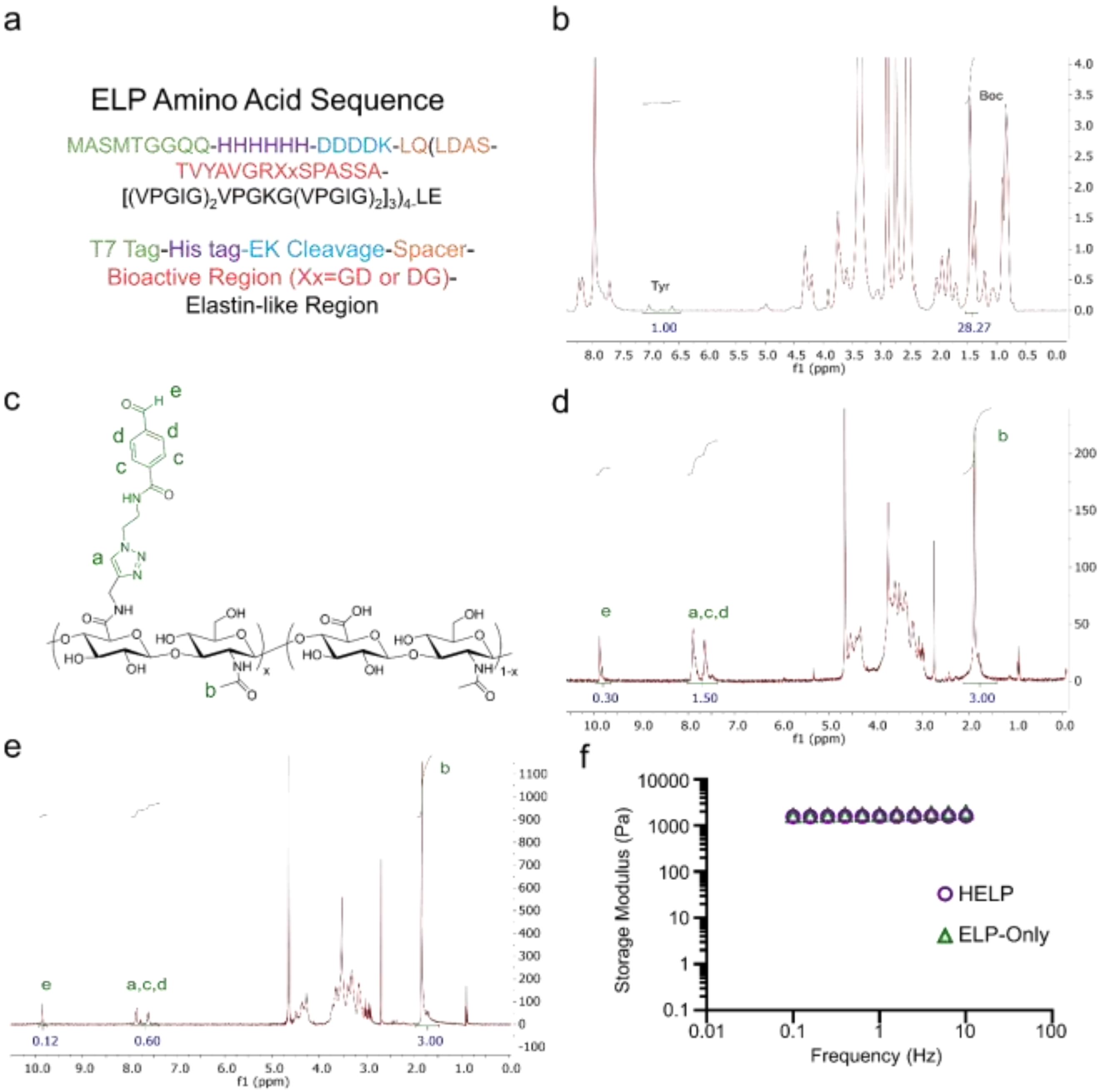

Figure S1: HELP protein backbone and chemical modification. a) ELP amino acid sequence that can be modified to contain the fibronectin-mimicking RGD or scrambled RDG motifs. b) Nuclear magnetic resonance (NMR) of ELP modified with a hydrazine moiety. c) Hyaluronan structure modified with a benzaldehyde moiety. d) NMR of 30% modified hyaluronan with corresponding peaks from c. e) NMR of 12% modified hyaluronan with corresponding peaks from c. f) Plateau shear storage moduli (G') of HELP and ELP matrices measured using oscillatory rheology.

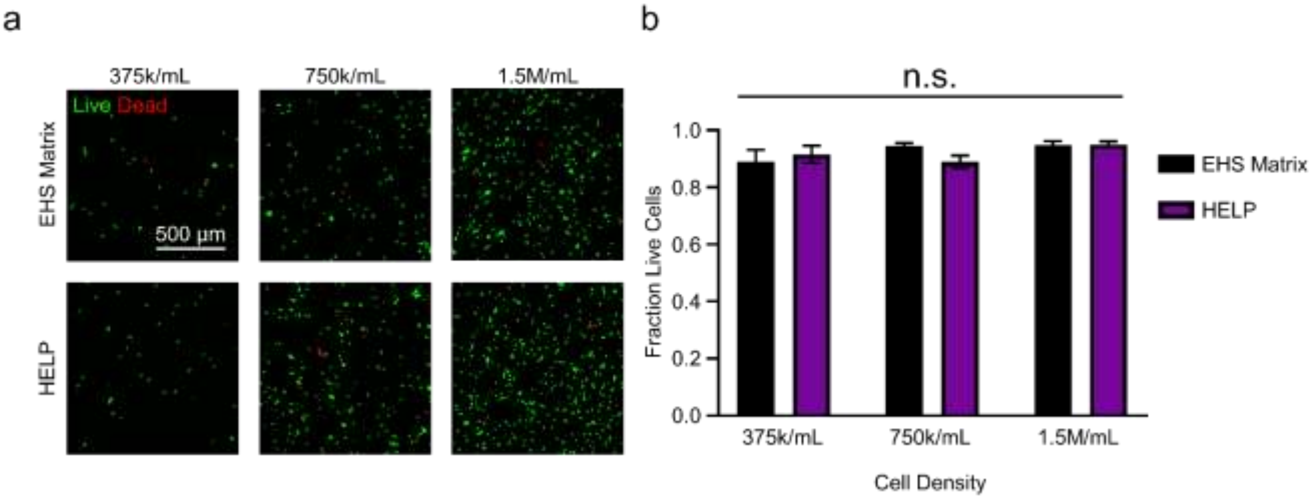

Figure S2: Cell viability upon 3D cell encapsulation. a) Fluorescence images of overlayed live (Calcein AM) and dead (ethidium homodimer) stains acutely post-encapsulation into EHS and HELP matrices over a range of cell densities. b) Quantification of cell viability, n=3, z-stacks of whole 6 µL hydrogels were taken, 2-way ANOVA, n.s. = [illegible]

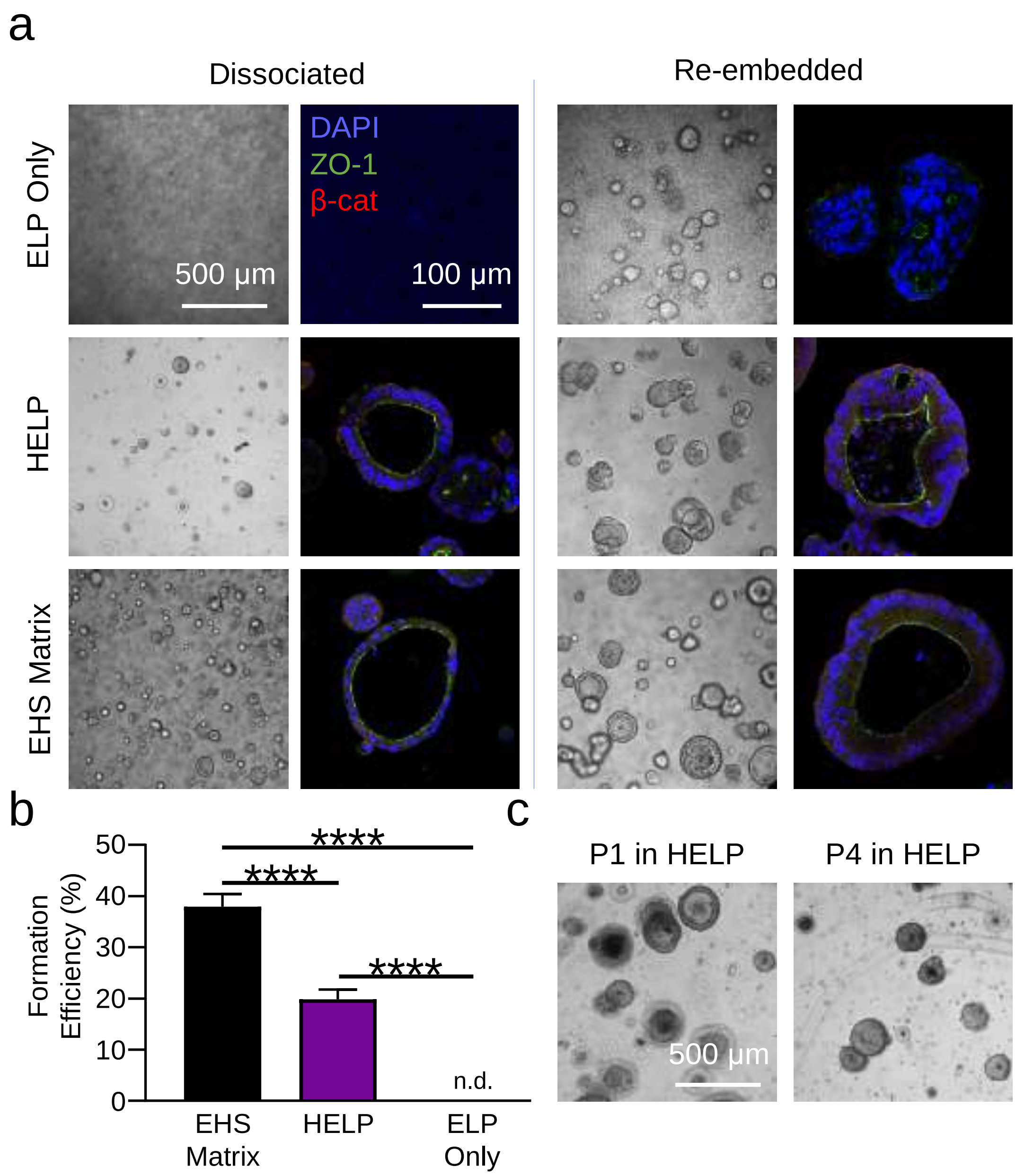

Figure S3: Human intestinal organoids robustly form in HELP in more than one patient-derived cell line. a) Brightfield and confocal fluorescence micrographs of spheroids in EHS, HELP, and ELP only matrices, when dissociated (left) and re-embedded (right). b) Spheroid formation efficiency of spheroids grown from single cells in the material formulations shown in a). 1-way ANOVA with Tukey's multiple comparisons testing, **** = $p<0.0001$, n=3, n.d. = none detected. Data shown are mean ± SD. c) Representative brightfield images of spheroids grown on Passage 1 and Passage 4 in HELP, with dissociation into single cells at the time of each passage.

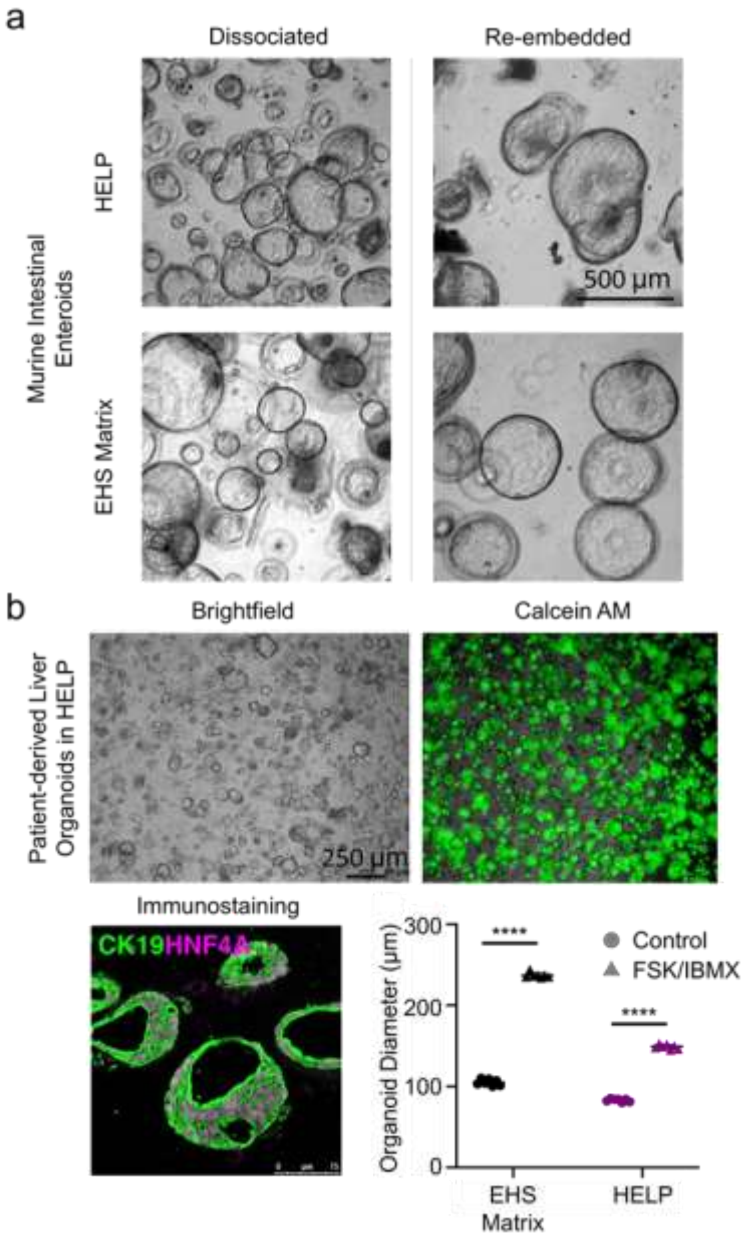

Figure S4: HELP broadly supports epithelial organoid formation and growth. a) Brightfield micrographs of murine small intestinal spheroids grown in HELP and EHS matrices, when dissociated (left) and re-embedded (right). b) Micrographs of human hepatic organoids in HELP, by brightfield (top left), Calcein AM fluorescence (top right) and immunostaining for cytokeratin-19 (CK19) and hepatocyte nuclear factor 4 alpha (HNF4A) (bottom left) . Quantification of human hepatic organoid size change in functional assay to show response to forskolin treatment (bottom right). 2-tailed Student's t-test, **** = $p<0.0001$, $n=7$. Data shown are mean ± SD.

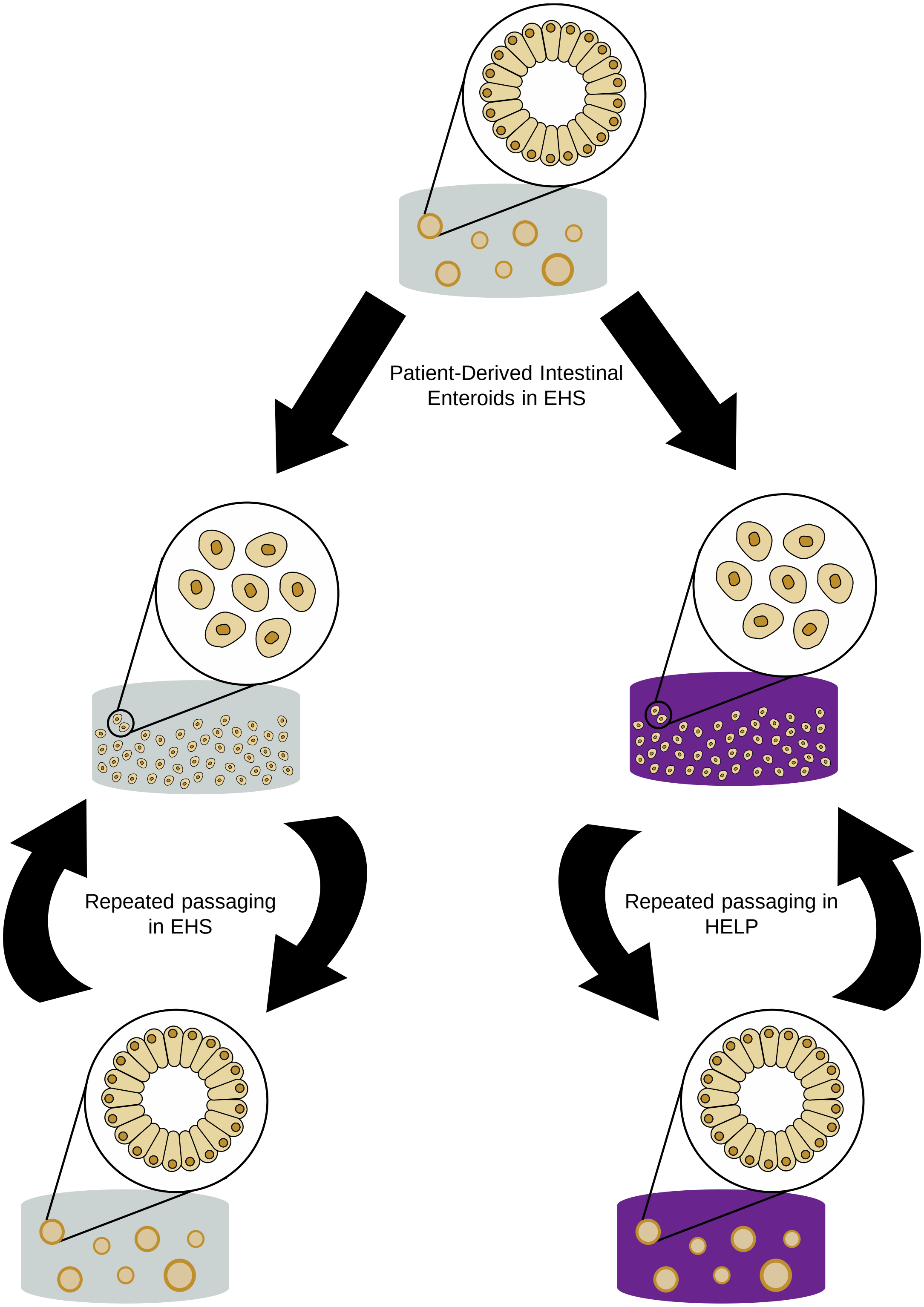

Figure S5: Intestinal organoids can be passaged within EHS or HELP matrices. a) Single patient-derived intestinal cells can be seeded in either EHS or HELP matrices and repeatedly passaged from single cells.

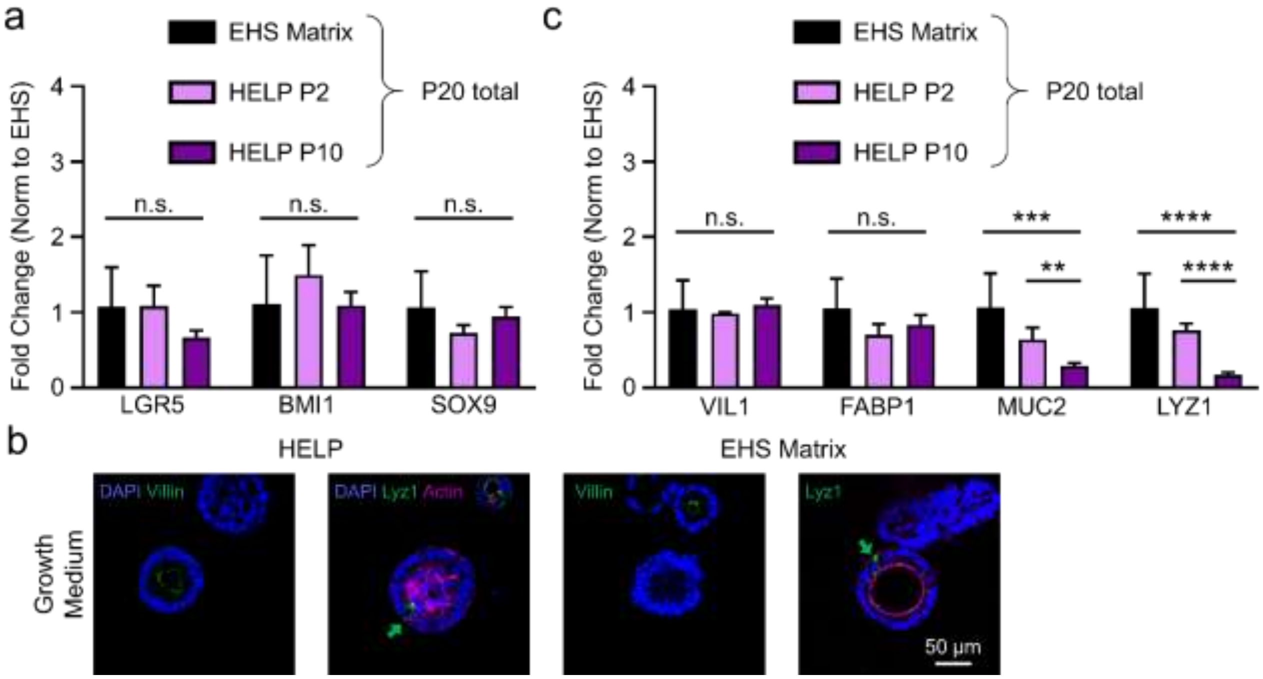

Figure S6: Expression of intestinal markers over time in HELP. a) Gene expression of intestinal crypt stemness markers LGR5, BMI1, and SOX9 for passage 20 enteroids maintained in EHS matrix for all 20 passages or in HELP for the final 2 or 10 passages (P2 and P10, respectively). All cultures were only exposed to growth medium throughout all passages. 1-way ANOVA, n=4, n.s. = not significant. b) Enteroids cultured in growth medium spontaneously express mature intestinal epithelial markers of enterocytes (Villin) and Paneth cells (Lysozme, Lyz1) in both HELP and EHS matrices. c) Gene expression of mature intestinal epithelial marker genes VIL1, FABP1, MUC2, and LYZ1 for the same cultures as in panel a). 1-way ANOVA with Tukey post-hoc testing, n=4, n.s. = not significant, ** = $p<0.01$, *** = $p<0.001$, **** = $p<0.0001$.

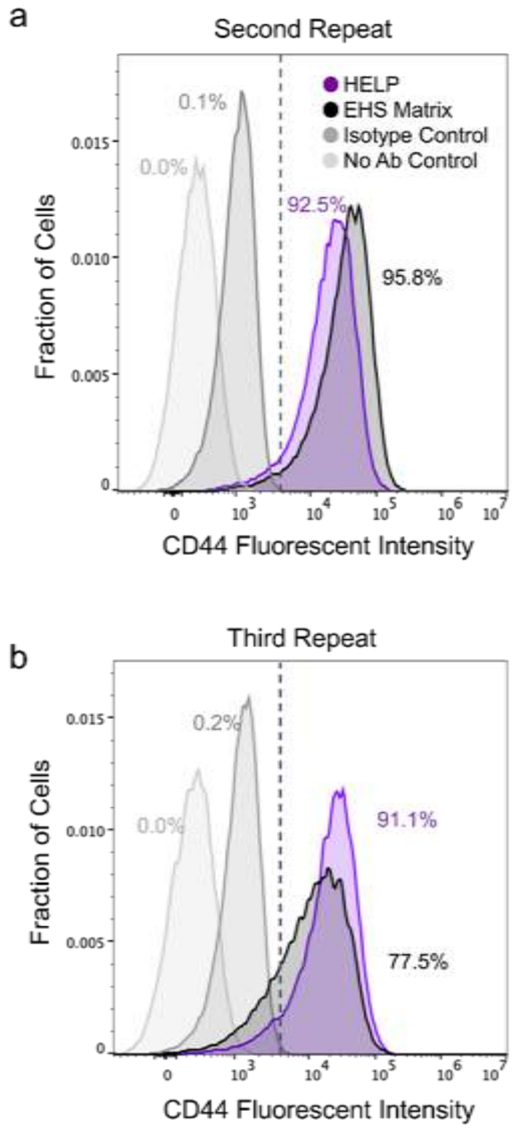

Figure S7: Flow cytometric analysis for CD44$^+$ cells in enteroids grown in HELP and EHS matrices. a) Second and b) third independent experimental repeats of flow cytometry data presented in Figure 3, showing the quantification of CD44$^+$ cells in each material, including negative controls.

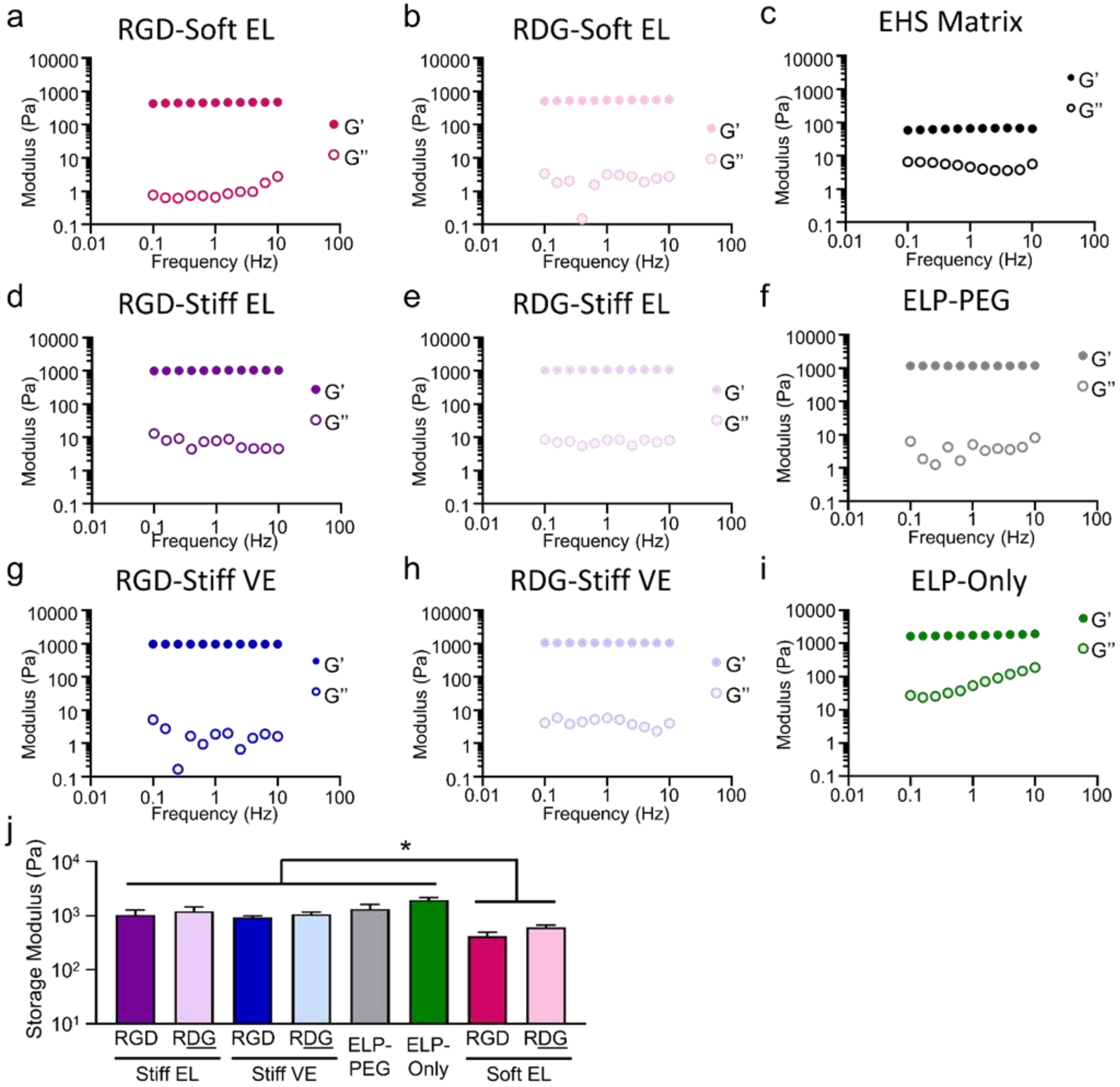

Figure S8: Material rheology. a-i) Oscillatory shear rheology was performed to characterize the viscoelastic properties of all hydrogel formulations reported in this work. These frequency sweeps were performed between 0.1-10 Hz at 1% strain, and the nominal stiffness of each matrix was determined based on the value of the storage modulus at 1 Hz. Data shown here are representative, with similar results obtained at least three times for each formulation. j) Summary data of storage moduli for formulations shown in a-i). At least 3 measurements were taken for each material. 1-way ANOVA with Tukey post-hoc testing, * = $p<0.05$. Data shown are mean ± SD.

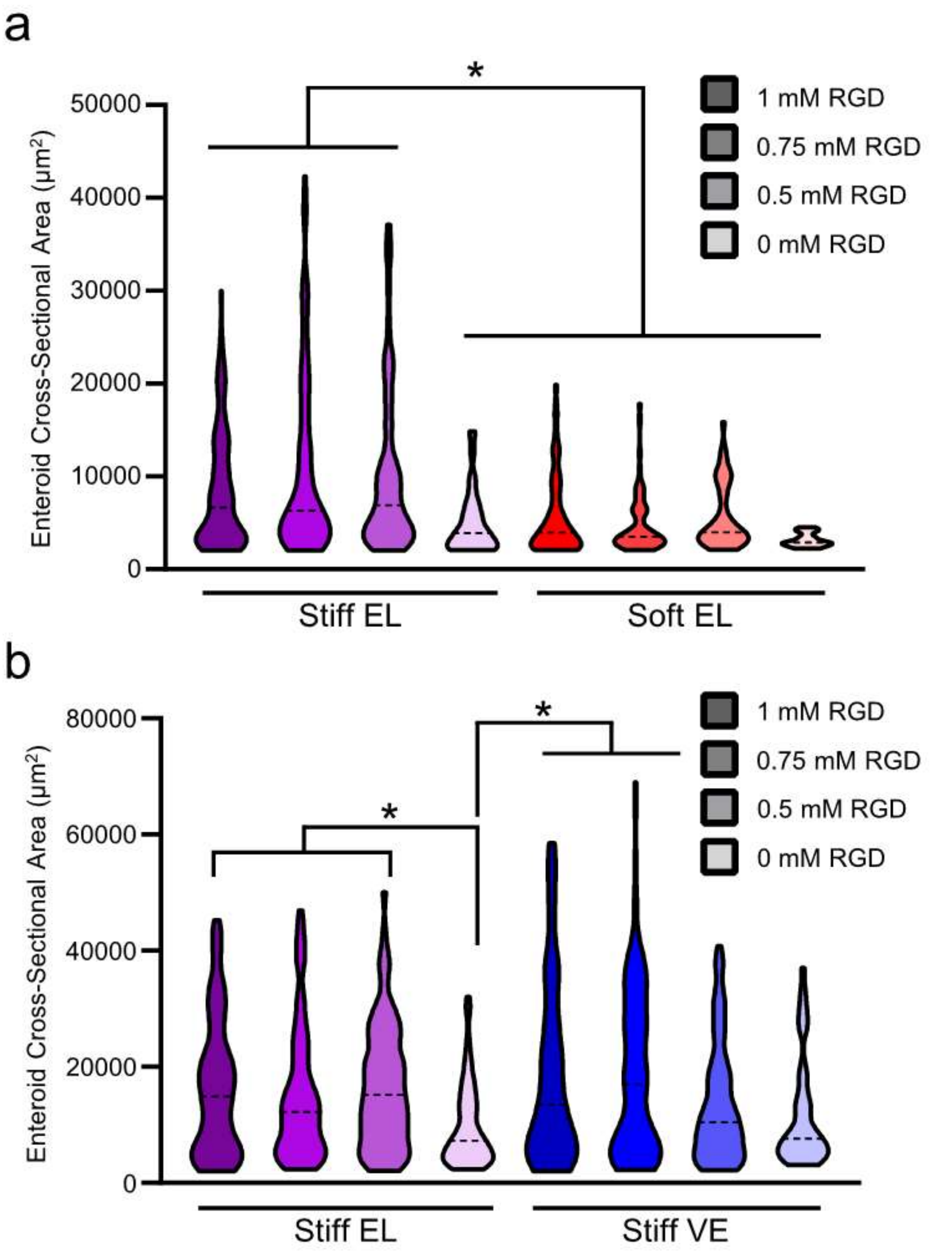

Figure S9: Cross-sectional area of spheroids grown in distinct HELP formulations. a) Cross-sectional area of spheroids grown in Stiff EL compared to Soft EL HELP with varying concentrations of RGD 12 days post-encapsulation. b) Cross-sectional area of spheroids grown in Stiff EL compared to Stiff VE HELP with varying concentrations of RGD 12 days post-encapsulation. Kruskal-Wallis test with Dunn's test for multiple comparisons. n=3 hydrogels per condition, * = $p<0.05$. Data shown are lumped distributions from technical replicates in each material.

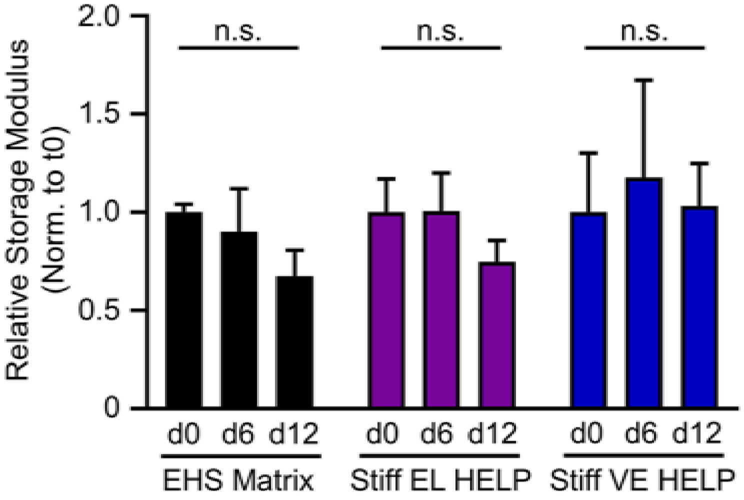

Figure S10: DLS microrheology of matrix mechanics over time. Human primary intestinal epithelial cells and polystyrene nanoparticles were encapsulated in hydrogels (EHS matrix, Stiff EL HELP, and Stiff VE HELP) and cast in cuvettes suitable for measurement on a dynamic light scattering (DLS) machine. Microrheological measurements were taken acutely, as well as at 6 and 12 days post-encapsulation in each material. 1-way ANOVA, n=4, n.s. = not significant. Data shown are mean ± SEM.

Table S1—Antibody Information

Target	Species	Vendor	Product #	Dilution
ZO-1	Mouse	Thermo Fisher	33-9100	1:150
β-catenin	Rabbit	Cell Signaling	8480	1:150
Lysozyme	Rabbit	Thermo Fisher	PA5-16668	1:100
Mucin-2	Rabbit	Santa Cruz Biotechnology	SC-15334	1;50
Chromogranin-A	Rabbit	Santa Cruz Biotechnology	SC-13090	1:50
CD44	Mouse	Santa Cruz Biotechnology	SC-7297	1:50
Ki67	Rabbit	Santa Cruz Biotechnology	SC-15402	1:100
HNF4α	Goat	Santa Cruz Biotechnology	SC-6556	1:100
Cytokeratin 19	Mouse	DAKO	M088801-2	1:50
Keratin 8	Rat	Dev. Studies Hybridoma Bank (DSHB)	TROMA-I	1:200

Table S2—PCR Primers

Target/GENE	Forward Primer (5'-3')	Reverse Primer (5'-3')
Villin-1/VIL1	CTGAGCGCCCAAGTCAAAG	AGCAGTCACCATCGAAGAAGC
Mucin-2/MUC2	GAGGGCAGAACCCGAAACC	GGCGAAGTTGTAGTCGCAGAG
Lysozyme/LYZ1	TCAATAGCCGCTACTGGTGTA	ATCACGGACAACCCTCTTTGC
Chromogranin A /CHGA	AGAATTTACTGAAGGAGCTCCAAG	TCCTCTCTTTTCTCCATAACATCC
β-actin/ACTB	CATGTACGTTGCTATCCAGGC	CTCCTTAATGTCACGCACGAT
